# Toxoplasma Encephalitis following Tandem Autologous Hematopoietic Stem Cell Transplantation: A Case Report and Review of the Literature

**DOI:** 10.1155/2018/9409121

**Published:** 2018-11-11

**Authors:** Vidya Kollu, Margarida Magalhaes-Silverman, Guido Tricot, Dilek Ince

**Affiliations:** ^1^University of Iowa, Department of Internal Medicine, Divisions of Hematology, Oncology, and Blood and Bone Marrow Transplant, Iowa City, IA, USA; ^2^University of Iowa, Department of Internal Medicine, Division of Infectious Diseases, Iowa City, IA, USA

## Abstract

Infection with *Toxoplasma gondii* is a rare but often fatal complication in hematopoietic stem cell transplantation (HSCT) recipients. Most cases have been reported in allogeneic (allo-) HSCT recipients, with only narrative reports following autologous HSCT (ASCT). We report the case of a 58-year-old Caucasian male presenting with toxoplasma encephalitis following tandem ASCT for myeloma and successfully treated with diagnosis by polymerase chain reaction analysis of cerebrospinal fluid. He was treated with sulfadiazine and pyrimethamine (with leucovorin) followed by pyrimethamine and atovaquone as secondary prophylaxis while receiving subsequent therapy for progressive multiple myeloma. Toxoplasmosis is a potential complication in allo-HSCT as well as ASCT recipients and should be considered in any post-HSCT patient with neurological dysfunction. Rapid diagnosis and immediate antimicrobial treatment are essential to avoid morbidity and mortality.

## 1. Introduction

Autologous HSCT (ASCT) is a pivotal therapeutic option for patients with multiple myeloma (MM) who are eligible for transplantation and achieve at least partial response after combination chemotherapy [1]. Tandem transplantation shows a higher overall survival and progression-free survival in MM when compared to patients who receive a single ASCT [2, 3]. Despite the many advances in supportive care, infections continue to complicate the posttransplant course in many patients [4].

Infection with *Toxoplasma gondii*, is a rare but often fatal opportunistic infection in HSCT recipients. Most cases have been reported following allogeneic (allo-) HSCT especially in patients with severe graft versus host disease, requiring intensive immunosuppressive therapy [5, 6]. However, there are reports of disseminated disease [7], retinochoroiditis [8], or solitary spinal cord lesion [9] following ASCT. Cerebral toxoplasmosis has been described in one case each following ASCT for non-Hodgkin's lymphoma [10], neuroblastoma in a pediatric patient [11], and after tandem ASCT for myeloma [12]. We report the case of a 58-year-old Caucasian male with toxoplasma encephalitis following tandem ASCT for multiple myeloma.

## 2. Case Report

### 2.1. Clinical Summary

A 58-year-old Caucasian male was diagnosed in early 2014 with multiple myeloma. He was treated with dexamethasone, cisplatin, doxorubicin, cyclophosphamide, and etoposide-induction chemotherapy and then tandem ASCT in June and September 2014, respectively. Pretransplant chemotherapy consisted of bortezomib, dexamethasone, thalidomide, and melphalan 200 mg/m^2^ (VDT-Mel). Prior to HSCT, the recipient was seropositive for *T. gondii*. Antimicrobial prophylaxis consisted of fluconazole, acyclovir, and ciprofloxacin. He developed low-grade fevers, headaches, and impaired memory following engraftment on day +17 after second transplant.

### 2.2. Diagnostic Workup

A new thalamic lesion with edema and mass effect on the third ventricle and faint peripheral enhancement was found on a contrasted brain magnetic resonance imaging (MRI). Blood cultures were negative. CSF (cerebrospinal fluid) demonstrated elevated protein, normal glucose, and a WBC count 94/*µ*L with predominantly lymphocytes (62) and histiocytes (32). Cytology showed no tumor cells. Cerebrospinal fluid (CSF) demonstrated elevated protein (245 mg/dl), normal glucose (48 mg/dl), and a WBC count 94/*µ*L with predominantly lymphocytes (62/*µ*L) and histiocytes (32/*µ*L). Cytology showed no tumor cells. CSF bacterial and fungal cultures, cryptococcal antigen assay, herpes simplex virus, enterovirus, human herpes virus 6, Ebstein‐Barr virus, and JC virus polymerase chain reactions (PCRs) were negative. HIV screen (antigen/antibody testing), fungal serologies, and Quantiferon-TB Gold were negative. CSF and serum toxoplasma PCR were positive. The patient was neutropenic (ANC < 500/*µ*L) for approximately seven days following both transplants, but lymphopenia lasted for a prolonged period.

### 2.3. Treatment

The patient was empirically started on sulfadiazine and pyrimethamine with leucovorin. He developed crystal nephropathy with renal failure while on sulfadiazine and was changed to clindamycin and pyrimethamine for 8 weeks. Response to treatment was favorable at 1-month follow-up, as assessed by clinical and radiological means. Subsequently, he was switched to pyrimethamine, leucovorin, and atovaquone as secondary prophylaxis for toxoplasma infection while he was lymphopenic during ongoing maintenance chemotherapy. The patient died 30 months after HSCT due to progressive myeloma with no evidence of toxoplasmosis.

## 3. Discussion

*Toxoplasma gondii* infection is usually a result of reactivation of latent infection rather than primary infection with rare exceptions [13]. Pre-HSCT seropositivity is an important risk factor for toxoplasma disease via reactivation in the posttransplant period [14], as in our patient. The markedly low lymphocyte count in our patient likely played a crucial role in the development of infection. Infection was likely a result of the lymphocytotoxic effect of corticosteroid which was a part of the induction and conditioning chemotherapy, the fact that tandem ASCT is more immunosuppressive than a single ASCT, and compromised immune recovery following transplantation.

### 3.1. Diagnosis

Diagnosing cerebral toxoplasmosis following HSCT is difficult and often necessitates a combination of radiologic techniques, PCR, and histology [15]. Direct detection of the parasite by visualization of tachyzoites by histopathology is a rare event. Diagnosis is most commonly made through detection by PCR [16].

PCR is the most valuable test and can be performed on blood, CSF [17, 18], or bronchoalveolar lavage samples, especially in untreated patients. Our patient was diagnosed by positive PCR, performed on serum and cerebrospinal fluid. It has an average sensitivity of 62%, which extends as high as 81% in untreated patients, but in previously treated patients, its sensitivity is only 20% [19]. While absolute diagnosis of toxoplasmosis continues to depend on biopsy and demonstration of the pathogen, PCR should be built into the diagnostic strategy before treatment is started, as it offers timely, valuable information and assists in the confirmatory diagnosis of toxoplasma encephalitis and ocular toxoplasmosis [20].

MRI is the most sensitive radiologic technique, typically displaying multiple lesions localized in the basal ganglia and the corticomedullary junction of the cerebral and cerebellar hemispheres [21]. Lesions often show central necrosis and are isointense or hypointense on T1 and hyperintense on T2-weighted images. Ring contrast enhancement is variable, depending on the patient's host inflammatory response and immune status with an inverse relationship with the leucocyte level, and it is reduced on corticosteroid treatment [22]. Our patient showed an atypical single lesion with only faint peripheral enhancement likely due to lymphocytopenia and corticosteroid use.

### 3.2. Treatment

Unlike immunocompetent individuals in whom the infection generally remains latent for life, in HSCT recipients latent toxoplasma infection can reactivate and, if inadequately treated or left untreated, can lead to a fatal outcome [23]. Preemptive treatment using routine blood PCR monitoring appears effective in detecting infection early and preventing disease, especially in seropositive allo-HSCT recipients and high-risk ASCT recipients when chemoprophylaxis is not possible [24]. The gold standard, both in the treatment of reactivation and disease, is the combination of pyrimethamine-sulfadiazine-leucovorin, while trimethoprim-sulfamethoxazole (TMP/SMX) is the agent of choice for primary prophylaxis in high-risk patients [25, 26].

Prophylaxis should ideally be started as soon as feasible after allo-HSCT but no later than engraftment and continued for at least six months, as about 10% of cases occur within one month and 90% of cases occur during within six months [26]. If engraftment is delayed and TMP/SMX cannot be given due to myelosuppression, atovaquone [27] can be used until engraftment, followed by TMP/SMX. A more controversial issue is the need for prophylaxis in ASCT recipients; such a policy is debatable due to the low incidence of toxoplasmosis in this setting and the consequent lack of experience.

## 4. Conclusion

Toxoplasmosis is an infrequent but serious complication following HSCT and requires a high index of suspicion to implement appropriate diagnostic tests and immediate treatment. Because reactivation is the usual mechanism of disease, determining a recipient's pretransplantation serological status for *T. gondii* is essential. Toxoplasmosis should be considered in the presence of neurological dysfunction even in the absence of typical radiological findings in pre-ASCT toxoplasma seropositive patients. Additional studies are essential to evaluate the efficacy of universal prophylaxis and to determine the role of secondary prophylaxis in high-risk ASCT seropositive patients.
